# Presenting complaints and mortality in a cohort of 22 000 adult emergency patients at a local hospital in Nepal

**DOI:** 10.7189/jogh.09.020403

**Published:** 2019-12

**Authors:** Samita Giri, Tormod Rogne, Oddvar Uleberg, Eva Skovlund, Sanu Krishna Shrestha, Rajendra Koju, Jan Kristian Damås, Erik Solligård, Kari R Risnes

**Affiliations:** 1 Department of Circulation and Medical Imaging, Norwegian University of Science and Technology, Trondheim, Norway; 2 Department of Community Programs, Dhulikhel Hospital, Kathmandu University Hospital, Dhulikhel, Nepal; 3 Department of Clinical and Molecular Medicine, Norwegian University of Science and Technology, Trondheim, Norway; 4 Gemini Center for Sepsis Research, St. Olav’s hospital, Trondheim University Hospital, Trondheim, Norway; 5 Department of Emergency Medicine and Pre-Hospital Services, St. Olav’s Hospital Trondheim University Hospital, Trondheim, Norway; 6 Department of Public Health and Nursing, Norwegian University of Science and Technology, NTNU, Trondheim, Norway; 7 Department of Emergency, Dhulikhel Hospital, Kathmandu University Hospital, Dhulikhel, Nepal; 8 Department of Internal Medicine, Dhulikhel Hospital, Kathmandu University Hospital, Dhulikhel, Nepal; 9 Department of Infectious Diseases, St. Olav’s Hospital, Trondheim University Hospital, Trondheim, Norway; 10 Clinic of Anesthesia and Intensive Care, St. Olav’s hospital, Trondheim University Hospital, Trondheim, Norway; 11 Childrens Clinic, St. Olav’s Hospital, Trondheim University Hospital, Trondheim, Norway

## Abstract

**Background:**

There is a need to develop sustainable emergency health care systems in low-resource settings, but data that analyses emergency health care needs in these settings are scarce. We aimed at assessing presenting complaints (PCs) and post-discharge mortality in a large emergency department population in Nepal.

**Methods:**

Characteristics of adult patients who entered the emergency department (ED) in a hospital in Nepal were prospectively recorded in the local emergency registry from September 2013 until December 2016. To assess post-ED mortality, patient households were followed-up by telephone interviews at 90 days.

**Results:**

In 21892 included adults, the major PC categories were injuries (29%), abdominal complaints (23%), and infections (16%). Median age was 40 years and sex distribution was balanced. Among 3793 patients followed at 90 days, 8% had died. For respiratory and cardiovascular PCs, 90-day mortality were 25% and 23%. The highest mortality was in individuals with known chronic lung disease, in this group 32% had died by 90 days of ED discharge, regardless of PC. In women, illiteracy compared to literacy (adjusted odds ratio (aOR) = 7.0, 95% confidence interval (CI) = 2.1-23.6) and being both exposed to tobacco-smoking and traditional cooking stove compared to no smoke (aOR = 2.8, 95% CI = 1.6-4.9) were associated with mortality. The mortality was much higher among family-initiated discharged patients (17%, aOR = 5.4, 95% CI = 3.3-8.9) compared to doctor-initiated discharged (3%).

**Conclusions:**

Our report suggests that nearly one in ten patients seeking emergency health care died within 90 days. This finding is alarming and novel. Post-discharge studies need to be replicated and appropriate follow-up programs in low-resource settings where primary health care is underdeveloped are urgently needed.

The Disease Control Priorities project has estimated that almost half of the deaths and over a third of the disabilities in low- and middle-income countries (LMICs) could be addressed through effective emergency care [1]. Top priority to emergency care has been recognized by the World Health Assembly [2]. A recent study reported that death rates and disability-adjusted life-years (DALYs) attributable to emergency conditions are three times higher in low-income countries (LICs) than high-income countries (HICs) [3]. Nevertheless, emergency health services are still underfunded and underdeveloped in LMICs [4], and it has been argued that improvement is particularly needed in emergency care systems [1,5]. However, a recent systematic review of emergency care in 59 LMICs has pinpointed the scarcity of relevant data that makes clinical and policy priorities difficult [6]. Another systematic review performed in 139 LMICs showed that patient outcomes from emergency care were poorly reported. There were 3-4 times more studies reporting mortality during emergency care compared to reporting outcomes after emergency discharge [7].

In Nepal, emergency care systems are underdeveloped [8,9]. Patients often directly access emergency care irrespective of the type of health complaints, and primary care physicians in emergency department (ED) are often the first contact point with health care. Previous studies from Nepal have focused on certain groups of emergency patients such as injuries and infections, and reported that patient volumes have increased in recent years [10-12]. A systematic review among traffic injuries in Nepal reported that the mortality rate had almost doubled from 2001 to 2013 [13], and that the burden of non-communicable diseases (NCDs) had almost doubled [14,15]. These reports, however, provide only fragments of the picture of emergency health care needs and understanding morbidity patterns may aid health administrators in resource allocation and planning of training needs [16,17]. Descriptive information about ED patients is scarce in Nepal. Surprisingly little is known about mortality after emergency care and studies in Nepal have reported hospital and ED mortality to one percent or less [18-20]. Mortality after ED visit is usually not documented, although discharge to home should not be regarded as a completion of patient management [21].

To add knowledge in this area, we aimed to describe 1) characteristics of adult ED patients across presenting complaints (PCs) in a hospital in Nepal; 2) mortality until 90 days after presentation and assess factors that are associated with mortality in this population. We took advantage of increasing access to mobile phones in Nepal and follow-up information was assessed by telephone interviews.

## MATERIALS AND METHODS

### Study design and setting

A prospective observational study was conducted in the ED of a non-government university hospital with 375 beds [22]. The hospital is located in semi-urban region in Dhulikhel, in Kavrepalanchok district 30 km northeast of Kathmandu. Kavrepalanchok district has a total population of nearly 400 000 and 51% are female [23]. The median age in this region is 23 years, and 30% are 0-15 years old. The corresponding figures for Nepal as a whole are: 23 years median age and 35% are less than 20 years. The three main ethnic groups in the district are Brahmin/Chheti (36%) followed by Janajati (51%) and Dalit (7%). The majority (78%) of the population in this district use wood as a main type of cooking fuel.

### Data collection and participants

Demographic and clinical information was prospectively registered in systematic emergency forms by ED nurses, paramedics and doctors, and was extracted into an electronic database by a research nurse (Appendix S1 in **Online Supplementary Document**). All adults (>16 years) presenting at the ED between September 2013 to December 2016 were included in the study. However, data collection was interrupted three times during the study period because of; failure to continue data collection (Sept 2014-Feb 2015), earthquakes (April 25-May 16 2015) and missing ED files (Sept and Nov 2016). Data from the earthquake period has been described previously [19].

### Variables

Research nurses used the patients’ home addresses to categorize their residence into rural (living outside a municipality) or urban (living inside a municipality). Ethnicity was categorized into four groups recognized by Nepali authorities; Brahmin and Chhetri, Janajati, Dalit, and others. Brahmin and Chhetri are generally considered as a group having a higher socioeconomic status and Dalit typically have a lower socioeconomic status [24].

Time of presentation at ED was categorized into; daytime (08-16 weekdays) and after working hours (16-08) or holidays. ED disposition was categorized into; hospitalized, non-hospitalized or dead in the ED. Hospitalizations were further categorized: admitted to general wards, directly transferred to ICU (Intensive Care Unit) or OT (Operating Theatre), or referred to other hospitals from ED. Non-hospitalized patients were categorized into; doctor-initiated discharge or family-initiated discharge (FID).

### Presenting complaints classification

**Figure 1:**
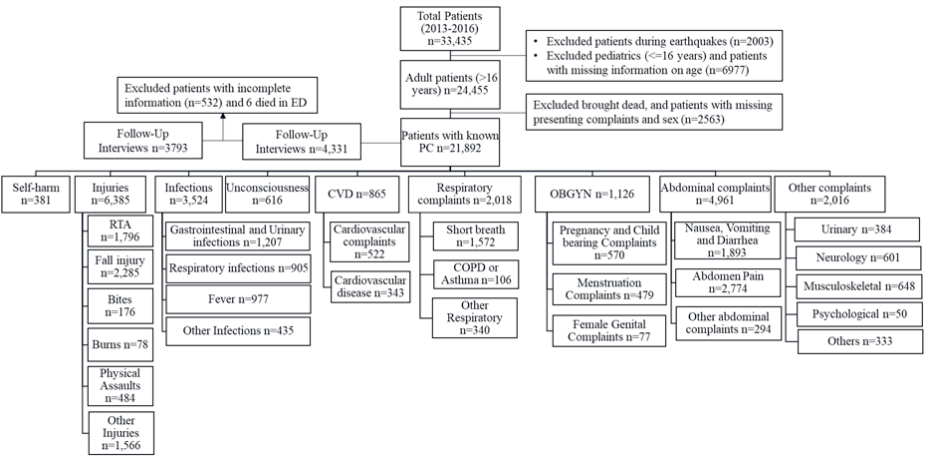
Flow diagram of cohort and distribution of presenting complaints. ED – emergency department, PC – presenting complaint, RTA – road traffic accidents, CVD-cardiovascular diseases or complaints, COPD – chronic obstructive pulmonary disease, OBGYN – obstetrics or gynecology.

The presenting complaints from the emergency forms were translated into “International Classification of Primary Care-2 (ICPC-2)” codes [25], and classified accordingly into nine main categories and each patient was assigned a primary PC category; self-harm, injuries, infections, unconsciousness, CVD (cardiovascular related complaints and diseases), respiratory complaints, OBGYN (obstetrics and gynecology), abdominal complaints and other complaints (**Figure 1**). Patients that had information on any NCDs at ED presentation in addition to the PC was given a NCD category; COPD (chronic obstructive pulmonary disease) or asthma, CVD and other NCDs (cancer, diabetes or chronic liver disease) in addition to the PC. The strategy for presenting complaint categorization and use of ICPC-2 codes is presented in the Annex S2 and Table S1 **Online Supplementary Document**.

### Follow-up interviews

At ED disposition all patients were asked for consent to be telephone-interviewed at 90 days after initial presentation to the ED, and trained research nurses called and interviewed the patient or a family member (Annex S2 in **Online Supplementary Document**). The telephone interview was structured, and questions were about death or hospitalizations during last 90 days, and demographic information (literacy, occupation, number of family members living together, exposure level to smoke) (Annex S3 in **Online Supplementary Document**).

### Data analysis

Descriptive data are presented by numbers and percentages. Associations between patient characteristics and mortality at 90 days were assessed by logistic regression models. Age and sex adjusted odds ratios (aORs) and unadjusted odds ratios (ORs) with 95% confidence intervals (CIs) are presented. Data analyses were performed using STATA 13.1 (StataCorp LP, College Station Texas, USA).

### Ethics

The study was approved by the institutional ethical review committee of Kathmandu University School of Medical Sciences in Nepal (58/13) and the Regional Committee for Medical and Health Research Ethics in South East Norway (2014/1246). As this study is based on routinely collected pseudo anonymized patient information in the hospital, informed consent from the patients was not obtained in individuals, as approved by the local ethical committee. Verbal consent was taken for information on telephone numbers, and at the beginning of the telephone interviews.

## RESULTS

### Patient characteristics

During the study period, 33435 patients were enrolled. In total 21892 patients were included in the analysis (**Figure 1**). The most common presenting complaints were injuries (29%), abdominal complaints (23%), and infections (16%) (**Table 1**). The median age of ED patients was 40 years (interquartile range IQR 26-60), and men and women were equally represented. Almost two thirds of the patients lived in rural areas.

**Table 1:** Baseline characteristics by categories of presenting complaints in 21 892 patients presenting to emergency department in Dhulikhel Hospital from Sept 2013- Dec 2016

Characteristics	Total	Injuries	Abdominal complaints	Infections*	Respiratory complaints	OBGYN	CVD	Unconsciousness	Self-harm	Other complaints†
**Total patients, n (%)**	**21892**	**6385 (29)**	**4961 (23)**	**3524 (16)**	**2018 (9)**	**1126 (5)**	**865 (4)**	**616 (3)**	**381 (2)**	**2016 (9)**
**Age, median (IQR)**	40 (26-60)	35 (25-50)	37 (26-53)	43 (26-62)	65 (52-73)	25 (21-30)	60 (42-71)	40 (25-60)	32 (23-45)	45 (28-64)
**Age (years), n (%):**										
7-45	12 220 (56)	4144 (65)	3041 (61)	1802 (51)	327 (16)	1051 (93)	238 (28)	339 (55)	278 (73)	1000 (50)
45-60	4125 (19)	1256 (20)	949 (19)	673 (19)	389 (19)	61 (6)	193 (22)	122 (20)	80 (21)	402 (20)
≥60	5547 (25)	985 (15)	971 (20)	1049 (30)	1302 (65)	14 (1)	434 (50)	155 (25)	23 (6)	614 (30)
**Female, n (%)**	11 365 (52)	2379 (37)	2809 (57)	2019 (57)	1037 (51)	1126 (100)	444 (51)	336 (55)	239 (63)	976 (48)
**Patient location, n(%):**										
Rural	13 150 (60)	4153(65)	2803 (57)	2062(58)	1149(57)	718(64)	424(49)	388(63)	250(66)	1203 (60)
Urban	7030 (32)	1663(26)	1833 (37)	1228 (35)	680 (34)	272 (24)	398 (46)	170 (28)	76 (20)	710 (35)
Information NA	1712 (8)	569 (9)	325 (6)	234 (7)	189 (9)	136 (12)	43 (5)	58 (9)	55 (14)	103 (5)
**Ethnicity, n (%):**										
Brahmin and Chhetri	9470 (43)	2627 (41)	2125 (43)	1625 (46)	907 (45)	517 (46)	372 (43)	261 (42)	135 (36)	901 (45)
Janajati	10 060 (46)	3001 (47)	2302 (46)	1566 (45)	930 (46)	466 (41)	412 (48)	277 (45)	183 (48)	923 (46)
Dalit	1798 (8)	565 (9)	380 (8)	249 (7)	149 (7)	123 (11)	67 (8)	65 (11)	54 (14)	146 (7)
Other	564 (3)	192 (3)	154 (3)	84 (2)	32 (2)	20 (2)	14 (2)	13 (2)	9 (2)	46 (2)

OBGYN – obstetrics or gynecology, CVD – cardiovascular diseases or complaints, IQR – inter-quartile range, NA – not available

*Infections and fever.

†Other complaints included musculoskeletal, neurology, urinary, psychology and other general complaints (Table S1 in **Online Supplementary Document**).

One third (35%) of the patients were hospitalized (**Table 2**). Of the 12101 (65%) non-hospitalized, 10% were FID (discharged by own or family’s wish although hospitalization was required according to medical evaluation), and 51% of FID were 17-45 years (**Figure S1** in the **Online Supplementary Document**). The reported overall mortality in the emergency department was very low (0.3%).

**Table 2:** Time of presentation and emergency department disposition presented by categories of presenting complaints among adults presenting to emergency department in Dhulikhel Hospital form Sept 2013-Dec 2016

Characteristics	Total	Injuries	Abdominal complaints	Infections*	Respiratory complaints	OBGYN	CVD	Unconsciousness	Self-harm	Other complaints †
**Total patients, n (%)**	**21892**	**6385 (29)**	**4961 (23)**	**3524 (16)**	**2018 (9)**	**1126 (5)**	**865 (4)**	**616 (3)**	**381 (2)**	**2016 (9)**
**Presentation to ED (n = 19 789)‡, n (%):**										
08:00-16:00 weekdays	7510 (38)	2056 (36)	1568 (35)	1279 (40)	884 (48)	315 (33)	349 (45)	233 (41)	109 (32)	717 (39)
16:00-08:00 or holidays	12 279 (62)	3659 (64)	2976 (65)	1938 (60)	945 (52)	640 (67)	434 (55)	335 (59)	229 (68)	1123 (61)
**ED disposition (n = 18 598)‡, n (%):**										
Non-hospitalized	12 101 (65)	3944 (70)	3045 (73)	1965 (66)	807 (48)	250 (29)	399 (58)	302 (59)	105 (31)	1284 (75)
Hospitalized	6429 (35)	1655 (30)	1145 (27)	1024 (34)	862 (51)	607 (71)	281 (41)	190 (37)	234 (68)	431 (25)
Died in ED	59 (0.3)	10 (0.2)	2 (0.04)	6 (0.2)	8 (0.4)	1 (0.1)	3 (0.4)	23 (4)	5 (1)	1 (0.1)
**Non-hospitalized (n = 12 101)‡, n (%):**										
Doctor-initiated discharge§	10 951 (90)	3615 (92)	2839 (93)	1817 (92)	667 (83)	188 (75)	334 (84)	241 (80)	59 (56)	1191 (93)
Family-initiated discharge‖	1150 (10)	329 (8)	206 (7)	148 (8)	140 (17)	62 (25)	65 (16)	61 (20)	46 (44)	93 (7)
**Hospitalized (n = 6429)‡, n(%):**										
General wards¶	4756 (74)	1171 (71)	839 (73)	840 (82)	703 (82)	596 (98)	178 (63)	80 (42)	44 (19)	305 (71)
ICU or OT	489 (8)	50 (3)	188 (17)	76 (7)	54 (6)	8 (1)	22 (8)	12 (6)	62 (27)	17 (4)
Reffered to other hospitals	1184 (18)	434 (26)	118 (10)	108 (11)	105 (12)	3 (0.5)	81 (29)	98 (52)	128 (55)	109 (25)

OBGYN – obstetrics or gynecology related complaints, CVD – cardiovascular diseases or complaints, ED – emergency department, ICU – intensive care unit, OT – operation theater.

*Infections and fever.

†Other complaints included musculoskeletal, neurology, urinary, psychology and other general complaints.

‡Analysis done with smaller denominators than the total shown at the top due to missing information.

§Discharge done by responsible doctor after completion of treatment.

‖Discharge requested by patient or family members against completion of medical treatment.

¶Admitted for observation and further treatment.

### Characteristics by presenting complaint categories

Among injuries, 65% were 17-45 years, and the majority (63%) were men (**Table 1****)**. Falls from heights were the most common cause of injury (36%) followed by traffic injuries (28%) (**Figure S2** in the **Online Supplementary Document**). Physical assaults accounted for 8% of injured patients, and 36% of these were women (**Table 1**). The majority of the patients (70%) in injury group were discharged from ED without further hospitalization (**Table 2**).

Infections accounted for 16% of the ED population, and women were slightly overrepresented in this group (57%) (**Table 1**). Respiratory and cardiovascular complaints accounted for 9% and 4% respectively and were similar distributed in both sexes. The proportion of patients with cardiovascular complaints that lived in urban areas was higher (46%) compared to the total ED population (32%). Patients with respiratory and cardiovascular complaints had higher hospitalization rates (51% and 41% respectively) than the average for the ED population (35%) (**Table 2**). Of the hospitalized patients with cardiovascular complaints, more than one in four were referred to other hospitals. The non-hospitalized patients with respiratory and cardiovascular complaints had higher FID rates (17% and 16% respectively) compared to 10% in the ED population.

Self-harm was the main PC for 381 patients (**Table 1**). The most notable findings in this group were the relatively low age and that women were over- represented; the majority (73%) of these patients were young (17-45 years) and the majority were women (63%). The Dalit ethnic groups were overrepresented among self-harm patients (14% vs 8% in the total ED population). The hospitalization rate was high (68%) and 27% needed ICU treatment. FID from emergency department was common (44%) in this group (**Table**).

### 90-day mortality

**Figure 2:**
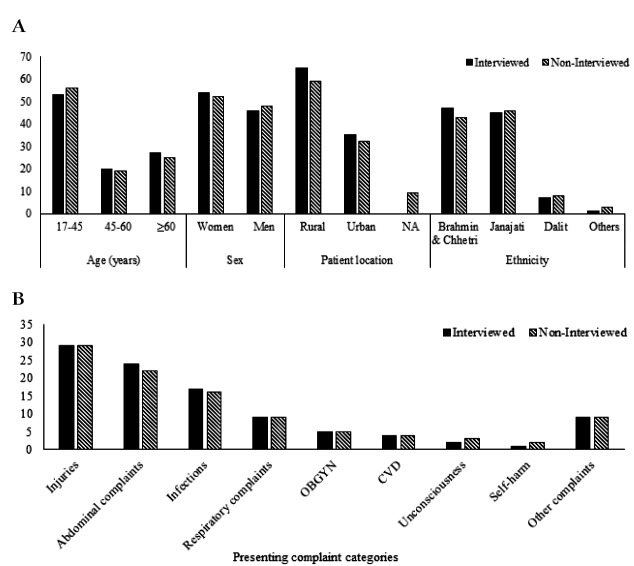
**Panel A.** Patient’s characteristics in the interviewed patients compared with non-interviewed patients. NA – information not available. **Panel B.** Presenting complaint categories in the interviewed compared with non-interviewed patients. OBGYN – obstetrics and gynecology, CVD – cardiovascular diseases and complaints.

Of the 21892 included patients, a total of 12540 household phone numbers were recorded in the patient registry. Of these 12540 patients, 4331 households (20% of total) participated in the structured telephone interview at 90 days (**Figure 1**). Among the interviews, we had complete information on 3793 (88%) patients and these were included in the further analysis. The patient demographic characteristics and presenting complaints in interviewed patients did, however, not meaningfully differ between those who were lost to follow-up (**Figure 2**, **panel A** and **Figure 2**, **panel B**).

Results for mortality at 90 days are presented in **Table 3**. The 90-day mortality in the cohort was 8% (n = 309), and mortality was higher in men (9%) compared to women (7%). The mortality was much higher in the older age group (23% in ≥60 years) compared to the younger groups (1% in 17-45 years). Compared to infections (7% 90-day mortality), corresponding mortality for injuries was 3% (aOR = 0.6, 95% CI = 0.4-1.0), for cardiovascular complaints 23% (aOR = 2.5, 95% CI = 1.5-4.1) and for respiratory complaints 25% (aOR = 2.4, 95% CI = 1.6-3.6). Patients who did not re-visit the hospital had higher mortality (10%) compared to those who had a second visit (7%), aOR = 1.4 (95% CI = 1.1-1.9). FID was strongly associated with mortality at 90 days (aOR = 5.4, 95% CI = 3.3-8.9) compared to doctor-initiated discharge. We assessed the associations of presenting complaints with 90 days mortality, taking NCDs into account. The results for these analyses show that mortality was particularly high for those with a NCD diagnosis, regardless of PC (COPD/asthma: 32%, CVD: 21% and other NCDs: 24%).

**Table 3:** Associations between presenting complaints and disposition characteristics, and 90 d mortality among 3793 patients interviewed by telephone after 90 d of emergency department visit

Characteristics	Total interviewed	90 d mortality	Unadjusted OR (95%CI)	Adjusted*OR (95%CI)
**Presenting complaints, n(%)**	**3793**	**309 (8)**		
Injuries	1085 (29)	34 (3)	0.4 (0.3-0.7)	0.6 (0.4-1.0)
Abdominal complaints	910 (24)	58 (6)	0.9 (0.6-1.3)	1.2 (0.8-1.8)
Infections	624 (17)	44 (7)	Ref	Ref
Respiratory complaints	343 (9)	87 (25)	4.5 (3.0-6.6)	2.4 (1.6-3.6)
OBGYN	200 (5)	0		
CVD	165 (4)	38 (23)	3.9 (2.5-6.3)	2.5 (1.5-4.1)
Unconsciousness	71 (2)	10 (14)	2.1 (1.0-4.5)	2.2 (1.0-4.8)
Selfharm	37 (1)	0		
Other complaints	358 (9)	38 (11)	1.6 (1.0-2.5)	1.5 (0.9-2.4)
**Presention to ED (n = 3561), n (%):†**				
08:00-16:00 weekdays	1527 (43)	163 (11)	Ref	Ref
16:00-08:00 and holidays	2034 (57)	123 (6)	0.5 (0.4-0.7)	0.7 (0.5-0.9)
**Hospital revisit, n (%):**				
Yes	2486 (66)	184 (7)	Ref	Ref
No	1307 (34)	125 (10)	1.3 (1.0-1.7)	1.4 (1.1-1.9)
**ED disposition (n = 3223), n (%):†**				
Non-hospitalized	1948 (60)	84 (4)	Ref	Ref
Hospitalized	1275 (40)	159 (12)	3.2 (2.4-4.2)	2.5 (1.9-3.3)
**Non-hospitalized (n = 1948), n (%):†**				
Doctor-initiated discharge‡	1744 (90)	50 (3)	Ref	Ref
Family-initiated discharge§	204 (10)	34 (17)	6.8 (4.3-10-8)	5.4 (3.3-8.9)
**Hospitalized (n = 1275), n (%):†**				
General wards‖	1037 (81)	122 (12)	Ref	Ref
ICU or OT	103 (8)	6 (6)	0.5 (0.2-1.1)	0.5 (0.2-1.3)
Referred to other hospitals	135 (11)	31 (23)	2.2 (1.4-3.5)	2.0 (1.3-3.3)

CI – confidence interval, OR – odds ratio, OBGYN – obstetrics or gynecology related complaints, CVD – cardiovascular diseases or complaints, ED – emergency department, ICU – intensive care unit, OT – operation theater

*Adjusted for sex and age in category.

†Analysis done with smaller denominators than the total shown at the top.

‡Discharge done by responsible doctor after completion of treatment.

§Discharge requested by patient or family members against completion of medical treatment.

‖Admitted for observation and further treatment.

Associations between patient characteristics, based on follow-up interviews, and 90-day mortality are presented in **Table 4**. Just above half of the patients were illiterate (53%) and the majority worked in own house or in agriculture (55%). The proportion exposed to smoke (tobacco and/or traditional stoves) was high (67%). There was no strong evidence of association between factors such as patient location, ethnicity, occupation or number of people in household and 90-day mortality.

**Table 4:** Associations between demographic factors and 90 d mortality among 3793 patients interviewed by telephone after 90 d of emergency department visit

Characteristics	Total interviewed	90 d mortality	Women (n = 2038)			Men (n = 1755)		
**90 d mortality**	**Unadjusted**	Adjusted*	**90 d mortality**	**Unadjusted**	Adjusted*
	**OR 95% CI**	**OR 95%CI**		**OR 95%CI**	**OR 95%CI**
**Total, n(%)**	**3793**	**309 (8)**	**143 (7)**			**166 (9)**		
**Age, (years), n (%):**								
17-45	2022 (53)	28 (1)	8 (1)	0.1 (0.04-0.2)		20 (2)	0.5 (0.3-1.0)	
45-60	755 (20)	43 (6)	27 (7)	Ref		16 (4)	Ref	
≥60	1016 (27)	238 (23)	108 (21)	3.5 (2.3-5.5)		130 (26)	8.0 (4.5-13.3)	
**Patient location, n (%):**								
Urban	1338 (35)	105 (8)	53 (7)	Ref	Ref	52 (9)	Ref	Ref
Rural	2455 (65)	204 (8)	90 (7)	1.0 (0.7-1.4)	1.0 (0.7-1.4)	114 (10)	1.1 (0.8-1.6)	1.2 (0.8-1.7)
**Ethnicity, n (%)**								
Brahmin and Chhetri	1772 (47)	143 (8)	68 (7)	Ref	Ref	75 (9)	Ref	Ref
Janajati	1690 (45)	140 (8)	63 (7)	1.0 (0.7-1.4)	0.9 (0.6-1.3)	77 (10)	1.0 (0.8-1.4)	1.1 (0.8-1.6)
Dalit	281 (7)	24 (9)	12 (8)	1.1 (0.6-2.1)	1.3 (0.7-2.7)	12 (9)	1.0 (0.5-1.9)	1.6 (0.8-3.2)
Others	50 (1)	2 (4)	0			2 (6)	0.6 (0.1-2.8)	1.5 (0.3-7.1)
**Education, n (%):**								
Literate	1765 (47)	48 (3)	3 (0.4)	Ref	Ref	45 (5)	Ref	Ref
Illiterate†	2028 (53)	261 (13)	140 (11)	33.9 (10.8-106.9)	7.0 (2.1-23.6)	121 (15)	3.7 (2.6-5.3)	1.0 (0.7-1.6)
**Occupation, n (%):**								
Paid job or business	933 (25)	56 (6)	10 (3)	Ref	Ref	46 (7)	Ref	Ref
Agriculture or housewives	2101 (55)	210 (10)	110 (8)	2.3 (1.2-4.5)	0.9 (0.4-1.9)	100 (15)	2.3 (1.6-3.2)	0.7 (0.4-1.0)
Student	264 (7)	1 (0.4)	1 (1)	0.2 (0.03-1.9)	1.2 (0.1-10.0)	0		
Elderly or sick‡	495 (13)	42 (8)	22 (11)	3.3 (1.5-7.1)	0.5 (0.2-1.1)	20 (7)	1.0 (0.6-1.7)	0.2 (0.1-0.3)
**No. of members in house, n (%):**								
≤5 members	2169 (57)	158 (7)	71 (6)	Ref	Ref	87 (9)	Ref	Ref
>5 members	1624 (43)	151 (9)	72 (8)	1.4 (1.0-2.0)	0.9 (0.7-1.4)	79 (11)	1.3 (0.9-1.7)	0.8 (0.6-1.2)
**Exposure to smoke, n (%):**								
None	1254 (33)	72 (6)	31 (4)	Ref	Ref	41 (8)	Ref	Ref
Traditional stove only§	1511 (40)	109 (7)	52 (6)	1.5 (0.9-2.3)	1.2 (0.7-1.9)	57 (9)	1.1 (0.7-1.7)	0.9 (0.6-1.4)
Tobacco only	559 (15)	62 (11)	26 (10)	2.4 (1.4-4.1)	1.8 (1.0-3.2)	36 (13)	1.7 (1.0-2.7)	1.5 (0.9-2.6)
Tobacco and traditional stove‖	469 (12)	66 (14)	34 (19)	5.2 (3.1-8.8)	2.8 (1.6-4.9)	32 (11)	1.5 (0.9-2.4)	1.4 (0.8-2.4)

OR – odds ratio, CI – confidence interval

*Adjusted for age in category.

†Interaction *P* < 0.0001.

‡Cannot work because of being old or sick.

§Traditional stoves do not have proper smoke outlet.

‖Interaction *P* = 0.09.

We assessed evidence of interactions by sex for associations between demographic factors and 90-day mortality. Thus, results for associations between demographic factors and 90-day mortality are presented separately for men and women. In women, literacy was strongly associated with 90-day mortality (aOR for illiteracy = 7.0, 95% CI = 2.1-23.6) compared to literate group (**Table 5****)**. No such association was found in men (interaction *P* < 0.001). In women, the association between exposure to smoke and 90-day mortality was strong: aORs for mortality in women exposed to traditional cooking stoves, tobacco smoking, and tobacco plus traditional cooking stove compared to no smoking exposure were 1.2 (95% CI = 0.7-1.9), 1.8 (95% CI = 1.0-3.2) and 2.8 (95% CI = 1.6-4.9) respectively.

**Table 5:** Associations between presenting complaints, including NCD information at presentation, and 90 d mortality among 3793 patients interviewed by telephone after 90 d of emergency department visit

Presenting complaints and NCD information, n (%)	Total interviewed	90 d mortality	Unadjusted OR (95%CI)	Adjusted* OR (95%CI)
Total	**3793**	**309 (8)**		
-Injuries, no NCD†	1048 (28)	24 (2)	0.5 (0.3-0.8)	0.6 (0.3-1.0)
-Infections, no NCD†	650 (17)	30 (5)	Ref	Ref
-Other PCs, no NCD*†	1583 (42)	119 (8)	1.7 (1.1-2.5)	1.7 (1.1-2.7)
-Any PC and COPD/Asthma	214 (6)	69 (32)	9.8 (6.2-15.7)	3.7 (2.3-6.0)
-Any PC and CVD	161 (4)	34 (21)	5.5 (3.3-9.4)	2.7 (1.6-4.8)
-Any PC and other NCDs	137 (4)	33 (24)	6.6 (3.8-11.2)	4.5 (2.5-7.9)

NCD – non communicable disease, CI – confidence interval, OR – odds ratio, PC – presenting complaints, COPD – chronic obstructive pulmonary disease, CVD – cardiovascular diseases. Other PCs – includes other presenting complaints such as; self-harm, obstetrics and gynecology, abdominal complaints, urinary, neurology, musculoskeletal and psychological. Other NCDs – include liver disease (n = 81), cancer (n = 19) and diabetes (n = 37).

*Adjusted for sex and age in category.

†Does not include NCD.

## DISCUSSION

### Population

Our study is in line with a systematic review in LMICs [6] and studies from Nepal [26] and Cambodia [27], showing that the majority of emergency patients are young adults. Injury was the main presenting complaint followed by abdominal complaints and infections, similar to reports from other LMICs [28,29].

Hospitalization rate from ED in the present study was lower (29%) than reported in other studies from Cambodia (60%) [27] and Pakistan (36%) [30]. This is probably due to different health care systems in these countries, and the fact that the study hospital receives unselected patients. Direct transfers from ED to ICU or OT were less frequent (8%) than reported in Pakistan (13%) [30]. However, these proportions do not depend only on severity, but also on the capacity of ICU and OT in the hospital. These observations indicate important variations in practice, and may complement the findings from a systematic review and reports from Nepal and Pakistan that reported a need for specialty trained ED providers [6], patient management protocols [8], availability of essential emergency equipment and knowledge among providers [31].

The rate of FID was high (10%). A study from India found this proportion to be (4%) [32] and in that study the majority were female and majority reported financial reason for the discharge request [32]. Sex differences for FID were not observed in our study, but over 50% were young (17-45 years), only one-third were ≥60 years. Information on reasons for FID was not available for this study, but based on local knowledge, it is often related to financial reasons, or a wish to continue medication at home. Especially in the elderly and severely ill population, terminal care at home is often preferred. In a study from USA, delay in care and inadequate patient-provider communication was the reason for FID [33]. We observed a very high 90-day mortality among patients after FID, much higher than in a population-based study in Manitoba [34]. The high mortality in this group can be explained by the Nepalese culture favoring dying at home. However, further investigations are required to understand reasons for FID or leaving hospital against medical advice.

### Injuries

Road traffic injuries in Sub-Saharan Africa and Southeast Asia have increased by 10%-50%, and are projected to be the sixth-leading cause of deaths and third-highest cause of DALYs in this region [4]. In Nepal, road traffic injury ranks 8th among the causes of premature deaths [35]. In the current study, the proportion of injury has increased compared to a report from the same hospital in 2013 [36]. We found fall injuries as the most common injury type, followed by traffic injuries. Young men were mostly affected by injuries, consistent with comparable settings [13,26,36-40]. The 90-day mortality in this group was 3%, a very high number when taking into account that the most severely injured patients may never have reached hospital. These findings indicate a need to establish robust trauma services and underline the importance of strengthening the health response capacity and health infrastructure in the rural regions. Moreover, prevention of injuries should be a national priority, and is achievable as evidenced from other developed countries in the past decades that has reported significant decrease in traffic related deaths [41].

### Self-harm

Although a relatively small proportion of ED patients, self-harm and suicide are increasingly recognized as a health problem in LICs, particularly in women. The maternal mortality and morbidity report from Nepal in 2008/09 had reported that suicide was the single leading cause of death among women of reproductive age (16%) compared to maternal related issues (12%) [42]. Suicide in Nepal is stigmatized and many could be reported as accidents. In the present study, more than two thirds of self-harm patients were women less than 45 years of age. In line with another study from Nepal [43], we found that these patients were seriously ill, and the majority were admitted to an ICU. These findings indicate that self-harm is a serious health problem especially among young women. Further studies and effective preventions are warranted.

### Mortality

Mortality by 90 days after emergency health care in the current study was more than 20-fold the ED mortality. The ED mortality in the current study was lower (0.3%) than previously reported from Pakistan (1.3%), and a systematic review in LMICs reported a median ED mortality of 1.8% (IQR 0.2%-5.1%) [6,44].

Very little evidence exists on mortality after emergency care in LMIC’s. To the best of our knowledge, only one study has previously attempted to assess mortality after emergency care. The study from a tertiary level Vietnamese hospital assessed 30-day mortality in two much smaller ED populations and reported mortality of 9.8% and 7.8%, respectively [45]. However, that study is different from ours since they did not include trauma and surgical cases, and the cohorts were recruited from a selected population in three months periods not taking into account seasonal variations [45].

Patients with respiratory and cardiovascular complaints had particularly high mortality. Thus, nearly one in four patients with these complaints died within 90-day. This is much higher than reports from HICs. The 60-day mortality among patients with respiratory complaints in a Spanish study was 6.3% [46]. Safwenberg and coworkers reported from a Swedish hospital that patients with cardiovascular complaints were at high risk for ten-year mortality (42% for chest pain and 67% for stroke-like symptoms), and suggested that the ED complaints are equally important as diagnosis in predicting long-term mortality [17].

Many factors may contribute to the high post-discharge mortality that we observed. We suspect that patients in the present study presented to health care at a late stage of chronic diseases, since health services are unaffordable for many of these patients [15,47]. Typically, availability of long-term treatments for COPD and CVD patients is very low, and follow-up systems for chronically ill patients are underdeveloped [15,48]. Also, local systems and transport systems that can handle quick and adequate responses to acute illness are underdeveloped, and contribute to a high post-discharge mortality in these patients. These results suggest a need to develop post-discharge care systems that would likely reduce long-term mortality in emergency patients.

Interestingly, we found illiteracy independently associated with increased mortality at 90 days in women but not in men. Secondary analysis of 2011 Nepal Demographic and Health Survey reports that illiterate Nepalese women are less aware of health risks, and that could result in less health seeking behavior [49]. Further, a higher mortality was observed with increasing dose of smoke exposure in women but not in men. A high burden of chronic lung disease in Nepalese women has been reported earlier, one study reported that COPD was nearly half of the NCD burden in Nepal [14]. Another study reported that the prevalence and incidence of COPD in men was high, but corresponding mortality and DALYs were higher in women [50]. Nepalese women typically spend much time cooking and many are exposed to traditional cooking stoves affecting respiratory health and symptoms of respiratory problems are often long-standing without seeking health care [51,52].

### Strengths and limitations

This is a single-center study, thus generalizations should be done with caution. However, the cohort comprised a large population from both rural and urban regions and the distribution of patient characteristics show that the patient population is highly representative for the region in respect to age, gender, geography and ethnicity. Also, the long data collection period is a strength; the study includes data from a three year period, covering possible seasonal variations. However, it is a limitation that the study had a low follow-up rate for the 90 days telephone interviews. For the interviews, the results should interpreted with caution, although we show that those lost to follow-up have similar characteristics and PCs. We cannot rule out the possibility of selection bias in the interviewed patients, leading to possible underestimation of mortality and a higher follow-up in healthier and more resourceful families. Patients who did not provide their telephone number were not interviewed and these patients might not have had a phone due to economic conditions, and could also be frailer than the ones available for interviews. Also, it is possible that those who were called and did not answer were more likely to have died.

Classification of presenting complaints was performed using a hierarchy approach which may result in an underestimation of frequency of complaints lower in the hierarchy (eg, if a patient had pneumonia and COPD then he/she would be allocated to the infection category).

## CONCLUSIONS

The study revealed that nearly one in ten died within 90-day of emergency care. However, follow-up rates were low and findings suggest a need for replication of post-discharge mortality studies with adequate systems that limit loss to follow-up. These studies should be performed as multi-center studies and include information on local health care systems that may affect post-discharge mortality. It will be important to study factors that affect post-discharge mortality, such as availability of follow-up systems, transportation and affordable medication. We argue that post-discharge mortality is a particularly important indicator on quality of care in low-resource settings, where primary care health systems are limited, and transportation and economic issues may halter adequate follow-up and treatment for complications or chronic diseases.

## Additional material

Online Supplementary Document
