# Supplementary material for: Presenting complaints and mortality in a cohort of 22 000 adult emergency patients at a local hospital in Nepal
Source: J Glob Health. 2019 Aug 25;9(2):020403. doi: 10.7189/jogh.09.020403 (PMC6708590; doi:10.7189/jogh.09.020403)

Presenting complaints and mortality in a cohort of 22,000 adult emergency patients at a local hospital in Nepal

Supplementary appendix, Table of contents

Annex S1 Emergency department registration

Annex S2: Presenting Complaints Classification

Annex S3 Follow-up interviews guide

Table S1: Categories of presenting complaints and use of ICPC-2 codes

Table S2: Characteristics of adult population presenting to emergency department before and after the earthquake during the study period

Table S3: Associations between patient's characteristics and mortality at 90 days before and after the earthquake

Fig S1: Distribution of types of injuries by sex

Fig S2: Distribution of family-initiated discharge by age and sex

Annex S1. List of variables included: Emergency Department registration/variables

1. Study Number
2. Hospital Number
3. Date of Admittance (DD/MM/YR)
4. Age (Years)
5. Sex (M/F)
6. Ethnicity
7. Time of Presentation
8. Temperature (Celsius)
9. Pulse (Beats per minute)
10. Respiration rate
11. Systolic Blood Pressure
12. Diastolic Blood Pressure
13. Oxygen Saturation
14. Glasgow Coma Scale (GCS)
15. Triage done (Yes/No)
16. Triage Code (Red/Orange/yellow/green)
17. Time of Triage
18. Time of Treatment
19. Presenting Complaint
20. Action in Emergency Room (Medication, Fluids, Oxygen, Antibiotics etc.)
21. Transfer to (General wards, ICU, OT)
22. ED disposition (Admitted, Discharged, LAMA/DOR, Referred)
23. Death (Yes/No)
24. Diagnosis at Discharge

Annex S2. Presenting Complaints Classification

The presenting complaints were recorded by an emergency nurse in the emergency form. The presenting complaint was recorded in free text format (for example; a lady with chest pain and fever). The complaints were retrospectively classified into nine main categories. The process of categorizations was performed in three phases using STATA software. In the first phase, in total 761"presenting complaint texts" (e.g. fever, chest pain, abdominal pain, limb fracture etc.) were generated and scored as 0 (no) or 1 (yes). In the second phase one or more "International Classification of Primary Care-2 (ICPC-2)" codes, were allocated to each patient based on the complaint categories first generated. In the third phase, one main presenting complaint was identified for each patient. Obvious related complaints were combined into one single group (e.g. fever and chills). The categories of nine main presenting complaints were; self-harm, injuries, infections, unconsciousness, CVD (cardiovascular related complaints and diseases), respiratory complaints, OBGYN (obstetrics and gynecology), abdominal complaints and other complaints. These categories were arranged in hierarchical order as shown in Fig 1 ("self-harm" at first and "other complaints" at last; i.e. if a patient had suicidal attempt irrespective to other complaints then he/she will be in self-harm category). Self-harm implicates attempted suicide; either poisoning or hanging. Infections complaint category included infections and also when presenting complaint was fever with no specification of organ system involved. The category "other complaints" included musculoskeletal, neurology, urinary, psychological and other complaints (general pain, feeling ill, male genital, eye/ear complaints etc.).

Follow-up interviews

Two trained research nurses conducted a structured telephone interview 90 days following the ED visit. The research nurse contacted all patients (n=12,540) who provided phone numbers during the ED disposition. The research nurse contacted twice when patients were not reachable the first time. The telephone interview included information on 90-days mortality, and demographics (literacy, occupation, number of family members living together, exposure level to smoke and hospital visit for the second time). Literacy was categorized as illiterate (no formal education) and literate (formal education). Occupation was categorized into; paid job or business, agriculture or housewives (women taking care of own house and family), student and elderly or sick (cannot work because of being old or sick). Exposure to smoke was categorized into four groups; none, traditional stove (no outlet for smoke coming out from the stoves), tobacco (cigarette or any kind of tobacco) and tobacco plus traditional stove.

Annex S3. Telephone interview guide: Follow-Up at 90 days after emergency department disposition

1. Study Number
2. Hospital Number
3. Date of Interview
4. Visited Dhulikhel Hospital (DH) for same condition after the last visit (Yes/No)
5. Visited other health facility for same condition after the last visit to DH (Yes/No)
6. How long did you wait in ED before seeing a doctor (in minutes)
7. How do you like the services/patient management provided from ED (Very poor, Poor, OK/Fair, Good, very Good)
8. Health status now (Better, Unchanged, Worse, Died)
9. If died, Date of dead (DD,MM,YR)
10. Information given by (Patient, Relatives, Others)

11\. a) In Labor case, if baby alive (Yes/No)

11\. b) Number of baby delivered

12\. Number of members in house living together

13\. Main occupation

14\. Education (Literate/Illiterate)

14\. Completed education level

15\. Smoking Tobacco (Yes/No)

15\. If smoking tobacco, number per day

16\. Fuel for Cooking (traditional cooking stove, improved cooking stove, gas stove, electricity)

17\. Additional Information

Table S1: Categories of presenting complaints and use of ICPC-2 codes

ICPC complaint Categories	Included ICPC-2 Codes
**Self harm**	P77
**Injuries**	
Fall Injury	A80_b*
Transport Accident	A80_a*
Physical Assault	Z25
Bite	A86, S12, S13
Burn	S14
Other Injury	A85, A88, N80, B77, D79, D80, R87, R88, X82, Y80, H76, H78, H79, F76, F79, A80_c*, A80_d*, L73, L74, L75, L76, L77, L78, L79, L80, L81, S16, S17, S18, S19
**Infections**	
Gastrointestinal/Urinary Infection	D70, U70, U71
Respiratory Infection	R74, R75, R76, R78, R81, R82, R83, A70
Fever (only)	A02, A03
Other Infection	A78, A87, B70, H71, N71, S11, S71, W70, W71, X72
**Unconsciousness & Seizure**	A06, N07
**Cardiovascular (CVD)**	
CVD complaints	A11/K01, K04, K05, K07
CVD disease	K71, K73, K75, K76, K77, K78, K84, K86, K90, K96
**Respiratory complaints**	
Short Breath	R02
COPD/Asthma	R95, R96
Other Respiratory	R03, R04, R05, R06, R07, R21, R24, R29, R84, R85, R99
**Obstretic & Gynaecology (OBGYN)**	
Pregnancy and Childbearing	W03, W05, W17, W18, W19, W27, W29, W80, W82, W83, W94, W96, W99
Menstruation Complaints	X02, X06, X07
Female Genital Complaints	X01, X14, X15, X18, X19, X21, X29, X87, X77, X99
**Abdomen and Digestive**	
Nausea, Vomiting & Diarrhoea	D09, D10, D11
Abdomen Pain	D01, D02, D03, D06, D12, D25, D88
Other Digestive	D04, D08, D13, D14, D15, D16, D17, D19, D20, D21, D24, D29, D74, D75, D76, D77, D78, D84, D85, D86, D89, D91, D95, D97, D98, D99
**Other Complaints**	
Urinary	U01, U02, U04, U05, U06, U07, U08, U14, U77, U88, U95, U99
Neurology	N01, N05, N06, N08, N17, N18, N19, N28, N29, N74, N85, N87, N88, N89, N99
Musculoskeletal	L01, L02, L03, L05, L07, L08, L09, L12, L13, L14, L15, L17, L18, L19, L29, L71, L82, L88
Psychosocial Complaints	P04, P06, P16, P19, P20, P29, P74, P76, P85, P86, P99
Other Complaints, NOS	A01, A04, A05, A08, A10, A29, A91, A92, B73, B82, F02, F05, F29, H01, H02, H04, S02, S06, S07, S29, S77, S91, T01, T03, T08, T11, T85, T86, T89, T90, T99, Y01, Y02, Y04, Y05, Y06, Y29, Y85, Y86, A66*

A80_b-Fall Injuries, A80_a-Road Transport Accidents, A80_c-Animal Injuries, A66-Referred to study hospital for intensive care unit.

*New codes that does not comply with ICPC-2 codes.

Table S2: Characteristics of adult population presenting to emergency department before and after the earthquake during the study period

Characteristics	Total	Before EQ	After EQ
**Total Patients, n (%)**	21892	7898 (36)	13994 (63)
**Age, median (IQR)**	40 (26-60)	40 (26-60)	40 (26-60)
**Age (years), n (%)**			
17-45	12220 (56)	4457 (56)	7763 (56)
45-60	4125 (19)	1432 (18)	2693 (19)
≥60	5547 (25)	2009 (26)	3538 (25)
**Female, n (%)**	11365 (52)	4241 (54)	7124 (51)
**Patient location n, (%)**			
Rural	13150 (60)	4367 (55)	8783 (63)
Urban	7030 (32)	2357 (30)	4673 (33)
Information NA	1712 (8)	1174 (15)	538 (4)
**Ethnicity, n (%)**			
Brahmin and Chhetri	9470 (43)	3571 (45)	5899 (42)
Janajati	10060 (46)	3545 (45)	6515 (47)
Dalit	1798 (8)	534 (7)	1264 (9)
Others	564 (3)	248 (3)	316 (2)
**ED disposition, n (%)**			
Non-Hospitalized	12101 (55)	3680 (47)	8421 (60)
Hospitalized	6429 (29)	3243 (41)	3186 (23)
Mortality at ED	59 (0.3)	7 (0.1)	52 (0.4)
Information NA	3303 (15)	968 (12)	2335 (17)
**Non-Hospitalized (n=12104) , n (%)**			
Doctor-initiated discharge	10951 (90)	3389 (92)	7562 (90)
Family-initiated discharge	1150 (10)	291 (8)	859 (10)
**Hospitalized (n=6437) , n (%)**			
General wards	4756 (74)	2563 (79)	2193 (69)
ICU or OT	489 (8)	272 (8)	217 (7)
Reffered to other hospitals	1184 (18)	408 (13)	776 (24)
**Presenting Complaints, n (%)**			
Injuries	6385 (29)	2182 (28)	4203 (30)
Abdominal complaints	4961 (23)	1763 (22)	3198 (23)
Infections	3524 (16)	1236 (16)	2288 (16)
Respiratory complaints	2018 (9)	808 (10)	1210 (9)
OBGYN	1126 (5)	590 (7)	536 (4)
CVD	865 (4)	234 (3)	631 (5)
Unconsciousness	616 (3)	223 (3)	393 (3)
Self-harm	381 (2)	166 (2)	215 (2)
Other complaints	2016 (9)	696 (9)	1320 (9)

EQ-earthquake, IQR-inter quartile range, NA-not available, ED-emergency department, ICU-intensive care unit, OT-Operational Theater, OBGYN-obstetrics and gynecology related complaints, CVD-cardiovascular diseases or complaints.

Figure S1: Distribution of family-initiated discharge by sex and age group in years

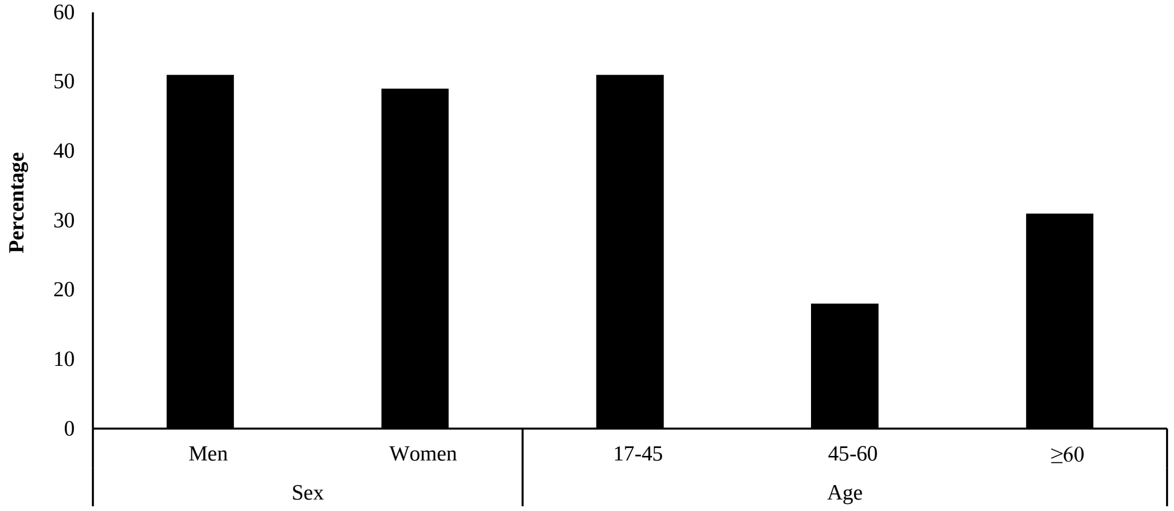

Figure S2: Distribution of types of injuries by sex

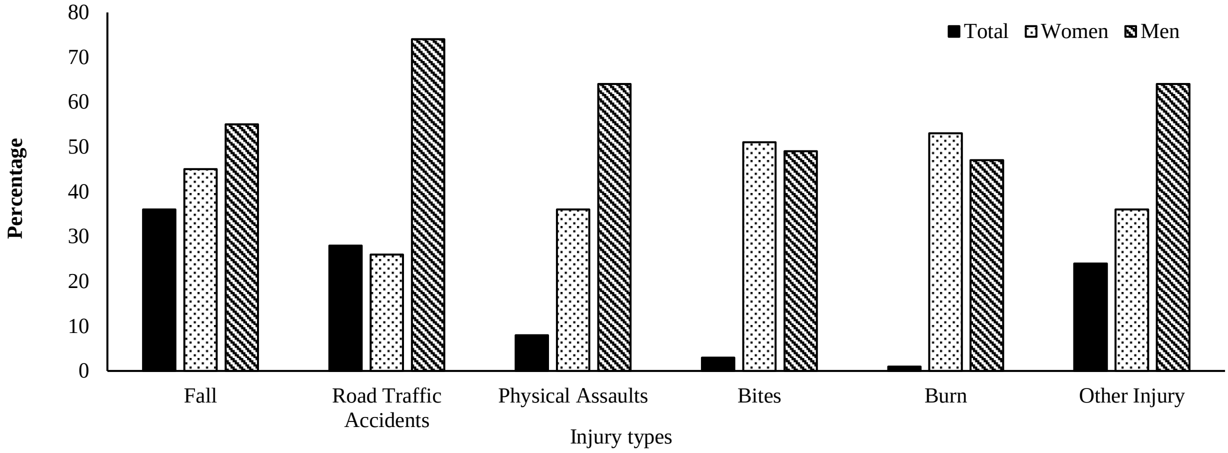

Supplement: Online Supplementary Document [file jogh-09-020403-s001.pdf]
